# Acute Renal Failure/Acute Kidney Injury (AKI) Associated with Endovascular Procedures

**DOI:** 10.3390/diagnostics10050274

**Published:** 2020-05-02

**Authors:** Zbigniew Krasinski, Beata Krasińska, Marta Olszewska, Krzysztof Pawlaczyk

**Affiliations:** 1 Department of Vascular, Endovascular Surgery, Angiology and Phlebology, Poznan University of Medical Sciences, 61-848 Poznan, Poland; zbigniew.krasinski@gmail.com; 2 Department of Hypertension, Angiology and Internal Disease, Poznan University of Medical Sciences, 61-848 Poznan, Poland; beata.bkrasinska@gmail.com; 3 Department of Nephrology, Transplantology and Internal Medicine, Poznan University of Medical Sciences, 60-355 Poznan, Poland; marta.olszewskakn@gmail.com

**Keywords:** acute kidney injury, endovascular procedures, contrast-induced nephropathy, risk factors, contrast media

## Abstract

AKI is one of the most common yet underdiagnosed postoperative complications that can occur after any type of surgery. Contrast-induced nephropathy (CIN) is still poorly defined and due to a wide range of confounding individual variables, its risk is difficult to determine. CIN mainly affects patients with underlying chronic kidney disease, diabetes, sepsis, heart failure, acute coronary syndrome and cardiogenic shock. Further research is necessary to better understand pathophysiology of contrast-induced AKI and consequent implementation of effective prevention and therapeutic strategies. Although many therapies have been tested to avoid CIN, the only potent preventative strategy involves aggressive fluid administration and reduction of contrast volume. Regardless of surgical technique—open or endovascular—perioperative AKI is associated with significant morbidity, mortality and cost. Endovascular procedures always require administration of a contrast media, which may cause acute tubular necrosis or renal vascular embolization leading to renal ischemia and as a consequence, contribute to increased number of post-operative AKIs.

## 1. Acute Kidney Injury

The kidney disease: improving global outcomes (KDIGO) group classification was proposed on the basis of the risk, injury, failure, loss of kidney function and end-stage kidney disease (RIFLE) 4 in 2012 [1,2,3,4]. Acute kidney injury (AKI) was defined as an increase of the serum creatinine (SCr) level exceeding 0.3 mg/dL within 48 h or an increase in SCr to 1.5 times the baseline value within 7 days or a urine output less than 0.5 mL/kg/hr for 6 h. KDIGO also classifies patients initiating renal replacement therapy (RRT) into stage 3 AKI, but removes the threshold of a 0.5 mg/dL increment for SCr > 4 mg/dL in the criteria of stage 3 AKI. The comparison of the definitions is shown in Table 1.

Contrast-induced nephropathy (CIN) is still poorly defined and its risk is difficult to determine due to a wide range of confounding individual variables. CIN has been traditionally defined by an absolute increase equal to or greater than 0.5 mg/dL (44 μmol/L) or an increase of at least 25% in serum creatinine levels sustained for two to five days in the absence of other identifiable causes starting within 48 to 72 h of the intravenous administration of an iodinated contrast agent [1,2,3,4]. CIN mainly affects patients with underlying chronic kidney disease (CKD), diabetes, sepsis, heart failure, acute coronary syndrome and cardiogenic shock. Moreover, it is associated with the progression of renal dysfunction, the requirement of dialysis, extended hospitalization, increased expenses and high mortality [4,5,6,7]. Many studies have shown an increased risk of AKI from CIN (Contrast-induced acute kidney injury-CIAKI). High-osmolar contrast media (CM) are unfavorable for routine intravascular injections due to high rates of adverse reactions, low biocompatibility and the biologic effects associated with hypertonicity [8]. Developed in 1974, next-generation low-osmolality CM are associated with fewer adverse reactions and improved tolerability. The osmolality is hypertonic when compared to average serum osmolality of 280–300 mOsm/kg, ranging from 780 to 800 mOsm/kg. In 1996, an iso-osmolar contrast iodixanol was developed. However, the use of it is reserved only for high-risk patients because of high cost and low risk of second-generation low-osmolality contrast agents nephrotoxicity in the general population (they are used in most scientific studies) [9]. CIAKI manifested as a sudden, but potentially reversible decline of glomerular filtration rate (GFR) typically occurs 48–72 h after CM infusion [7]. The pathogenesis of CIAKI is heterogeneous and, thus, is not entirely understood [7]. Three basic mechanisms appear at the same time for CIAKI development: A) renal vasoconstriction and B) medullary hypoxia, tubular cell toxicity and C) reactive oxygen species formation. The relative contribution of each of these mechanisms is unclear, but they eventually lead to epithelial and endothelial cell apoptosis and consequently GFR reduction.

AKI is one of the most common albeit underdiagnosed postoperative complication which may occur after any type of surgery. Despite the progression of surgical techniques (including endovascular procedures) its incidence is rising proportionally to the number of performed operations. Notwithstanding the advancement of endovascular methods, classic ion contrast is widely used nevertheless, posing a greater risk of AKI in intra-arterial interventions as it requires higher doses of iodized agents than in diagnostic CT–angio. Analysis of Centers of Medicare and Medicaid Services data demonstrate that between 1996 and 2011, the rate of lower extremity endovascular interventions increased from 138 to 584 interventions per 100,000 patients [10]. Contrast administration during endovascular diagnostic or therapeutic procedures is one of the major causes of AKI and the third leading cause of hospital acquired renal failure in the United States. Over 50% of these cases are the result of contrast exposure during cardiac catheterization, however, this number does not include post-contrast AKI following angiography or endovascular interventions for peripheral artery disease (PAD) [11].

## 2. Contrast-Induced Nephropathy

Nephrotoxicity has been observed in many cases of iodinated contrast use, however, the definition of CIN is still unclear. Fang et al. in 1980 symptoms such as oliguria, increased serum creatinine and decreases in fractional excretion of urinary sodium [12]. Depending on the definition adopted for CIN and the characteristics of the studied population, the incidence of CIN ranges from 10% to 30% [13,14,15,16,17]. Some authors have found that CIN is the third most common cause of hospital-acquired AKI [13]. Historically, the diagnosis of CIN and CIAKI have been confused; while CIN presupposes causality, CIAKI may signify CIN or AKI concomitant to the use of iodinated contrast instead of being caused by it [18]. Most published clinical studies use the two terms interchangeably and do not include control groups in their series, which raises questions concerning the actual incidence of CIN as well as the acute and chronic impacts on renal function introduced by the use of iodinated CM [18]. Therefore, any change in serum creatinine level that meets the defined threshold for post-contrast AKI is diagnosed as CIN. In some groups of patients, AKI can lead to permanent as well as increased morbidity and mortality [13,17]. Only a few studies have assessed the increased risk of CIN following intravenous administration, and most studies have not included a control group of patients who were not exposed to intravascular CM [19]. Davenport et al. claimed that intravenous low-osmolality iodinated contrast material is an important independent risk factor for post-computer tomography (CT) AKI, but not in patients with a stable serum creatinine level lower than 1.5 mg/dL (132.60 mmoL/L) [20]. This relationship strengthens with increasing pre-CT serum creatinine [20]. Newhouse et al. analyzed over 3081 publications, but only 40 of them (1.3%) included patients who received contrast intravenously [21]. Many of these studies were simply observational or retrospective studies, obtaining data from medical databases. These studies include confounding variables, such as sepsis, critical illness requiring intensive care, pre-existing diabetes, hypertension, CKD or cardiovascular disease [9]. Should be noted that the manner of contrast administration, such as intravenous vs. intra-arterial and volume (dose) of contrast used are also risk factors for CIN. There is much controversy in medical literature regarding the incidence of CIN [22]. A recent publication that addresses CIN risk by Wilhelm–Leen et al. used administrative data obtained from a 2009 subset of an inpatient care database with 8 million hospitalizations per year [22]. The study contained data from a cohort of 5.9 million that included many disease states, such as congestive heart failure, infection, acute pancreatitis and gastrointestinal bleeding. The patients who received iodinated contrast were matched with those who did not receive contrast and the risk of AKI was determined by statistical analysis. The risk of AKI was also determined when disease states were stratified. The authors found that the risk of AKI in patients receiving and those not receiving radiocontrast was nearly identical (5.5% vs. 5.6%, respectively). Data from disease-specific strata revealed that the risk of AKI was lower in those with acute coronary syndrome. This may be explained by the fact that those deemed to be at the highest risk of AKI are treated in order to minimize the risk. In cases of pancreatitis, the risk of AKI doubles with contrast exposure, but this increase may be attributed to other causes. The rates of AKI, death and dialysis within 30 days of imaging were relatively similar to previous reports and did not differ significantly between the contrast and non-contrast groups. The authors assumed that the risk of CIN may be lower than anticipated by clinicians, but they also admitted that the relationship “between radiocontrast administration and AKI is highly confounded, unpredictable and sometimes bidirectional” [22]. Clinicians admit that the risk of CIN is “likely low, but not likely zero” [22]. On the other hand, there are many other factors that contribute to the development of post-CT AKI, and not all cases of post-CT AKI are due to CIN [20]. These factors likely account for the equivalence in post-CT AKI rates after unenhanced and contrast-enhanced CT shown in some studies [23,24,25]. If these factors are not controlled, it is impossible to isolate the role of contrast material in the development of post-CT AKI [20].

## 3. Risk Factors of Post-Contrast AKI

The main risk factors for AKI include coexisting: a) diabetic nephropathy, b) advanced age (>75 years), c) congestive heart failure, d) volume depletion, e) hyperuricemia, f) proteinuria and g) high or recurrent exposure to CM. Concomitant use of NSAIDs, ACE inhibitors, diuretics or other nephrotoxic agents may further increase that risk. Among patients undergoing vascular surgery, AKI rates range from 5.9% after endovascular renal surgery, 12.7% for lower limb surgery and up to 48% for aortic arch surgery [26,27,28]. The incidence of AKI following major vascular surgery using the current criteria has been reported to be as high as 49% across a cohort of all vascular surgery patients, though there are significant differences depending on the type of procedure performed [29]. Thoracic and abdominal aortic procedures (endovascular as well as open surgical repair (OSR)) carry a higher risk of AKI than peripheral vascular operations. Peripheral vascular procedures have some of the lowest rates, from 4% for patients undergoing infrainguinal lower extremity bypass, up to 19% for endovascular revascularization of critical limb ischemia [26,30,31,32].

It seems that the other factors, related to endovascular surgery itself, e.g., vascular area of the vessel/its diameter—(aorta vs. limb arteries, thoracic aorta and arch vs. abdominal aorta), complexity of the procedure/duration of the procedure (simple stent grafts vs. branched or fenestrated devices) (Table 2), acute vs. planned procedures, critical procedures anemia vs. stop claudication, contribute substantially to the development of AKI [33,34,35,36,37,38]. Preoperative risk assessment and optimal perioperative management can minimize postoperative complications, e.g., AKI. Adherence to a standardized perioperative pathway designed to reduce risk of AKI after major vascular surgeries offers a promising clinical approach to mitigate the incidence and severity of this challenging clinical problem [36].

## 4. Coronary Angiography or Angioplasty

AKI is a common complication (Table 3) after diagnostic and therapeutic coronary procedures and is independently associated with adverse outcomes [39]. It has been shown that intra-arterial compared with intravenous CM administration may be linked to a greater risk of CIN, although the mechanism of this phenomenon is not clear [40,41]. The development of AKI in patients with ST-segment–elevation myocardial infarction undergoing percutaneous coronary intervention (PCI) is mainly related to older age, baseline eGFR, heart failure and hemodynamic instability [42]. However risk of AKI is similar between ST-segment–elevation myocardial infarction patients with and without contrast medium exposure [42]. Patients undergoing coronary angiography and PCI present an increased risk of CIN especially in the setting of urgent intervention [5,6], at which time high doses of CM are usually administered and a strong relationship between contrast volume and a risk of CIN is noticeable [5,6].

Therefore, invasive cardiologists ought to seek anatomic landmarks (particularly calcifications) to navigate their interventions without contrast usage, during both angiography and PCI. Furthermore, low volume injections with subsequent evacuation of unused contrast from catheters may improve patients’ outcomes and therefore must become a standard procedure [39]. Adequate hydration with intravenous saline infusion before and after intervention are key preventive approaches with regard to CIN [4].

AKI is a common complication occurring in the ∼600,000 patients who undergo PCI in the United States annually [51]. Despite improvements in PCI safety and outcomes, post-PCI contrast-induced AKI, remains common, occurring in almost 7% of patients [6,52,53]. Further, AKI is an independent risk factor for mortality [54,55,56,57,58] and nonrenal complications such as bleeding, sepsis [59] nonfatal myocardial infarction and stroke [60,61,62]. More direct complications of AKI include volume overload, hyperkalemia and progression to ESRD [6,51,63].

Transcatheter aortic valve implantation (TAVI) has become an established method for treating severe symptomatic aortic stenosis in patients who are poor candidates for open surgical procedure. As such, patients admitted for TAVI have considerable comorbidities making them especially vulnerable to a large number of periprocedural and postprocedural complications. In previous studies, up to 66% of patients who underwent the procedure experienced at least a moderate reduction in renal function [64,65,66]. Following TAVI, a further decrease in renal function commonly occurs, affecting 15%–40% of all patients and has proven to be predictive of several-fold increase in both short-term and long-term mortality [65,67,68,69]. The definition of worsening renal function has varied over time, albeit, the most commonly used definition for patients undergoing valve implantations is currently the Valve Academic Research Consortium-2 (VARC-2) criteria, in which a mild (26 mmoL/L) increase in creatinine within seven days identifies patients with AKI [70,71].

Recently, new strategies dedicated to the reduction of contrast volume have been proposed, and these appear to be promising in the battle against CIN [72,73,74,75]. Finally, wider use of intravascular imaging techniques (especially IVUS) should decrease injected contrast volume and improve PCI results [39]. Recently, Azzalini et al. published results of multicenter registry which included patients with chronic total occlusion who underwent PCI. CIAKI was defined as an increase in serum creatinine ≥ 0.3 mg/dL or ≥ 50% from baseline within 72 h [76]. Study endpoints were CIAKI, and all-cause death and target-lesion failure (cardiac death, target-vessel myocardial infarction or target-lesion revascularization) on follow-up [76]. Study population included 1092 patients (CKD *n* = 214, no CKD *n* = 878). Patients with CKD had more comorbidities as well as adverse angiographic features compared with subjects without CKD [76]. Patients with CKD experienced lower technical (79% vs. 87%, *p* = 0.001) and procedural (79% vs. 86%, *p* = 0.008) success rates. CI-AKI developed in 9.1% (CKD 15.0% vs. no CKD 7.8%, *p* = 0.001) [76]. Rates of in-hospital need for dialysis were 0.5% vs. 0%, respectively (*p* = 0.03). Patients with CKD had higher 24-month rates of all-cause death (11.2% vs. 2.7%, *p* < 0.001) and new need for dialysis (1.1% vs. 0.1%, *p* = 0.03), but similar TLF rates (12.4% vs. 10.5%, *p* = 0.47) [76]. CIAKI was not an independent predictor of all-cause death or target-lesion failure [76]. In the last study, Azzalini et al. included 111 patients (ultra-low contrast PCI group (UL-PCI), *n* = 8; conventional group, *n* = 103) [77]. Contrast volume (8.8 mL; interquartile range, 1.3–18.5) vs. 90 mL (interquartile range, 58–140 mL); *p* < 0.001) was markedly lower in the UL-PCI group [77]. Technical success was achieved in all UL-PCI procedures; in seven out of eight cases (88%), the UL-PCI protocol was also successful (contrast volume to eGFR ratio < 1) [77]. The incidence of CIAKI was 0% vs. 15.5% in the UL-PCI and conventional groups, respectively (*p* = 0.28). An ultra-low contrast PCI protocol in patients with advanced CKD is feasible, appears to be safe and has the potential to decrease the incidence of CIAKI, compared with angiographic guidance alone [77]. Sacha et al. presented a new concept for the use of the zero-contrast approach is the protection of residual renal function in hemodialysis patients undergoing coronary interventions [78,79]. Zero-PCI was feasible in each intended patient, including those with complex left main stenosis or lesion within a saphenous vein graft, and there was no specific complication associated with this technique [79]. After the procedure, the factual AKI prevalence was 10% and no patient required renal replacement therapy [79]. Three out of four hemodialysis patients preserved their residual renal function [79]. During the median follow-up of 3.2 (1.2–5.3) months no patient experienced an acute coronary event or required revascularization [79]. The residual renal function is a prognostic and independent factor of quality of life, morbidity and survival in dialysis patients and therefore every protective measure to preserve this function is important [80,81,82].

## 5. Endovascular and Surgical Procedures Related to Aorta

Due to a significantly different risk of AKI depending on a procedure, endovascular procedures were divided into aortic and peripheral for the purposes of this analysis. Thoracic and abdominal aortic procedures are related to higher risk of AKI than peripheral vascular operations. Complications specific to endovascular aneurysm repair include contrast nephropathy and renal ischemia secondary to endograft malpositioning or migration [36]. Elective endovascular aortic repair (EVAR) of infrarenal abdominal aortic aneurysms (AAA) have been related to incidences of AKI between 5.5% and 18% [37,38], although a study was published in which rate was as low as 2.9%, however it is to note that the definition of AKI used in that study, differed from KDIGO consensus criteria and was identified by increased serum creatinine of 0.5 mg/dL or a new dialysis requirement [83]. More complex AAA repairs have higher rates of AKI, up to 28% for those requiring branched or fenestrated devices, and as high as 32% for juxtarenal AAAs employing a snorkel or chimney approach [84,85]. Similarly, the recently reported incidence of AKI after thoracic endovascular aortic repair (TEVAR) for thoracic aortic aneurysms (TAA) was 9.7% while after repair of Stanford Type B acute aortic dissections amounted to 30% [33,34]. It should be emphasized that the common judgment regarding increased frequency of kidney injury after endovascular aneurysm repair (EVAR) vs. open surgery (OR) has not been confirmed by EBM operative tests. Open aortic procedures have significantly higher incidences of AKI compared to endovascular approaches [37]. Regardless of surgical technique (open or endovascular) this risk is escalated for more proximal aneurysms. Emergent repair of a ruptured aneurysm has the highest risk of AKI with some series documenting rates of 75% [35,86]. Open thoracic repairs had similarly elevated incidence of renal complications compared to their endovascular counterparts where AKI rates can be 34% for elective TAA, 45% for Stanford Type A dissections and 48% for aortic arch procedures [33,34,35]. Patients undergoing elective OR of infrarenal AAAs have AKI rates of up to 26% [37]. This risk is rising for more proximal aneurysms, up to 47% for juxtarenal and 68% for suprarenal aneurysms [35].

In the Amsterdam Acute Aneurysm Trial, the necessity for either temporary or permanent dialysis was observed significantly less often after EVAR than after OR [87]. In the study by van Beek et al., the incidence of AKI defined by the RIFLE criteria, was lower after EVAR (63%; 95% CI, 51%–74%) than after OR (76%; 95% CI, 71%–81%) [86]. This confirms that AKI occurred more often after OR than EVAR. Open thoracic repairs had similarly elevated incidence of renal complications compared to their endovascular counterparts where AKI rates can be 34% for elective TAA, 45% for Stanford Type A dissections and 48% for aortic arch procedures [27,33,88]. In the case of EVAR in the abdominal section, the stengraft fastening system play important role. This can be secondary to direct trauma to the renal arteries with suprarenal fixation barbs or dissection of the renal artery during the repositioning of a mal-deployed aortic endograft with suprarenal barbs [38,89,90,91,92].

In meta-analysis of 25 non-randomized studies comparing suprarenal (SR) with infrarenal (IR) fixation of 54,832 patients, a small, but significant difference in outcomes for renal dysfunction at the study end point was found (SR 5.98% vs. IR 4.83%; odds ratio (OR) 1.29, 95% confidence interval (CI) 1.18–1.40 (*p* < 0.001)) [89]. AKI has been reported in just under 20% of patients undergoing elective EVAR, and is worse in patients with poor cardiovascular reserve, with this decline remaining for over a year. Furthermore, more than a quarter of patients developed severe AKI after EVAR for ruptured AAA [93,94,95,96]. In one series comparing open and endovascular repair of ruptured AAAs, the incidence of AKI was 36%, with a higher incidence of AKI and mortality seen in the group treated with open surgical repair [96]. The US National Inpatient Sample (NIS) noted significantly less frequent acute renal failure for EVAR (12.1%) compared with 19.6% in OSR. In Medicare patients, EVAR was associated with 33.4% incidence of acute renal failure despite contrast administration versus 45.4% for OSR [97]. However, it is unclear whether the EVAR procedure itself or the cardiovascular risk profile of the patient and/or re-interventions are affecting long-term renal function. Compared with open aneurysm repair, EVAR is associated with fewer operative hemodynamic changes that have an impact on renal function. However, EVAR is accompanied by other potential hazards to the kidneys, such as exposure to CM and microemboli [98]. Reduced renal function and poor cardiovascular reserve have been described as significant risk factors for AKI after EVAR [94,99].

Operations associated with a high risk of AKI include ruptured AAA. Extensive blood loss, hypoperfusion and shock can affect the onset of AKI, irrespectively. Other influencing factors include age and general atherosclerosis, hypovolemic state, aortic cross-clamping, use of potentially nephrotoxic medications and CM [100,101]. Previous studies report an incidence of AKI ranging between 20% and 34% [100,102,103]. In a study conducted at three hospitals in Amsterdam as part of the Amsterdam Acute Aneurysm Trial between 2004–2011 on a group of 362 patients the incidence of acute kidney injury (AKI) defined by RIFLE criteria (‘risk,’ ‘injury,’ ‘failure,’ ‘loss,’ and ‘end-stage’) and according to the Society for Vascular Surgery/International Society for CardioVascular Surgery (SVS/ISCVS) reporting standards were measured. Regardless of assessment method, AKI occurred more often after open surgery compared with EVAR, 76% vs. 63% (RIFLE) and 50% vs. 40%, respectively (SVS/ISCVS reporting standards) [87]. It is unquestionable that the emergent repair of a ruptured aneurysm has the highest risk with some series documenting rates of 75% [88,104]. Renal injury after EVAR for rAAA ranges from 5% to 23% and is lower than renal failure after open surgical repair, which occurs in 16%–35% of cases [102,105,106,107,108]. A study on a limited number of ruptured aortic aneurysms treated with stengraft has shown that replacing iodine CM with CO_2_ may be beneficial for perioperative kidney damage prevention [109]. The study by Sailer et al. retrospective evaluation of renal function changes after intra-arterial administration of high volume of low-osmolar iodinated CM in a large patient population was observed [84]. The study covered large intravascular reconstructive procedures of 157 patients who underwent fenestrated and branched EVAR. Twenty-eight percent of patients developed post-EVAR AKI. In this study long procedure and time of occlusion of accessory renal arteries were independent risk factors for the development of AKI (odds ratio (OR) 1.005 per minute, 95% CI 1.001–1.01; *p* = 0.025 and OR 3.02, 95% CI 1.19–8.16; *p* = 0.029). Post-EVAR AKI was associated with a significantly increased risk of eGFR decline at discharge and later follow-up (hazard ratio (HR) 3.47, 95% CI 1.63–7.36, *p* = 0.001 and HR 3.01, 95% CI 1.56–5.80; *p* = 0.001). In this study iodinated contrast volume was not an independent risk factor for AKI or a decrease of eGFR during follow-up.

Multiple factors contribute to renal injury after EVAR. A study by Saratzis et al. considered clinical, anatomical and peri-operative factors in comprehensive evaluation of potential risk factors of AKI. An analysis of a cohort of 947 patients undergoing elective EVAR demonstrated that reduced pre-operative renal function was the main factor associated with AKI [99]. Sailer et al. in retrospective study confirmed that iodinated contrast volume was associated with a slightly increased risk for every milliliter of contrast applied; however, it was not an independent risk factor for AKI and long-term renal function decrease post-EVAR in complex aneurysms [84]. Although univariate analysis suggested a significant effect of contrast volume for the development of AKI, this effect subsided after adjusting procedure time. This finding corroborates the hypothesis that high contrast volume is a marker for the complexity of the procedure and that patients undergoing more complex procedures have a higher risk for renal function decrease [110]. In the mid- and long-term renal function observation presence of AKI was a significant predictor of eGFR decline at discharge and follow-up, the data provide no evidence that procedure time or other factors were significantly associated with eGFR decrease beyond 48 h. Although no significant associations with the other risk factors were found, directions of the hazard ratios conform to expectations of older age, diabetes and baseline eGFR [84].

Accessory renal artery coverage and other iatrogenic renal artery side branch occlusions with consecutive visible parenchymal perfusion defect on angiogram or follow-up CTA was a significant predictor for eGFR decrease in the immediate post-operative period. Any new parenchymal perfusion defect identified on angiogram or CTA is therefore an indicator of short-term decrease of renal function. At discharge and later follow-up, this risk factor was still associated with increased risk of eGFR diminishment (HR = 1.970 and HR = 1.371, respectively), but this association was no longer statistically significant (*p* = 0.106 and *p* = 0.384, respectively). This finding is in line with the results from a non-randomized retrospective study, where no significant long-term renal function deterioration was found in patients with accessory renal artery coverage during EVAR compared to a control group [111]. Although pre-operative increased creatinine levels appeared to play a role in the present cohort, this was not confirmed for renal function based on eGFR. It can only be speculated that routine pre-operative nephrological consultation and pre-operative hydration in patients with lower eGFR may have blunted the effect of decreased renal function in the present series [112]. In one randomized study analyzing the effectiveness of hydration in patients at risk of contrast induced nephropathy (patients with an eGFR of 30–59 mL/minute/1.73 m^2^) undergoing an elective procedure requiring iodinated contrast administration a difference between intravenous 0.9% NaCl hydration versus no hydration has not been found [113,114]. Thus, the potential benefit of hydration in patients with reduced kidney function undergoing EVAR is not clear and should be further evaluated.

## 6. Peripheral Vascular Intervention

The continuous development of endovascular surgery and increasing use in peripheral vascular intervention (PVI) method in managing different peripheral vascular diseases should direct our awareness to the incidence of AKI related to those procedures (Table 4), its risk factors and predictors of long-term consequences of AKI in this group of patients. In the meta-analysis by Prasad et al. analyzed 65 studies which included 11,311 patients with 10,316 peripheral procedures performed, where median incidence of AKI was 10% [115]. Authors highlighted significant variations in patient risk factors, definitions of AKI and specificity of description of endovascular therapies. In patients with peripheral vascular disease, there is often ischemic renal disease and preoperative reduction in eGFR is the strongest predictor of post-operative AKI. Administration of radiocontrast during endovascular procedures for peripheral arterial disease (PAD) may cause AKI.

Al Adas et al. analyzed AKI in patients after peripheral interventions. Only patients who developed AKI, which was defined as an increase in serum creatinine concentration of > 0.5 mg/dL within 30 days after intervention, were included (881 pts) [122]. In this group, 57 patients (6.5%) developed AKI; 47% were male and 51% had baseline CKD. AKI resolved by discharge in 30 patients (53%). Using multivariate linear regression, male sex (*p* = 0.027) and congestive heart failure (*p* = 0.048) were associated with 1-year GFR decline. Periprocedural variables related to 1-year GFR decline included percentage increase in 30-day post procedural creatinine concentration (*p* = 0.025), whereas CIN resolution by discharge (mean, 13.1 days) was protective for renal function at 1-year observation (*p* = 0.02) [122]. Retrospective observational cohort study by Sigterman et al. includes all patients who newly presented to the vascular surgery outpatient clinic with Rutherford class II or III PAD and who were treated with either supervised exercise therapy or endovascular interventions [123]. Changes in eGFR after one year were compared between the two treatment groups. Authors showed that after one year, eGFR was reduced by 8.6 mL/min (95% confidence interval [CI], 7.3–9.9, *p* < 0.001) in endovascular intervention group (*n* = 284) and by 1.7 mL/min (95% CI, 0.9–25, *p* < 0.001) in supervised exercise therapy group (*n* = 299) [123]. After correction of potential confounders, endovascular interventions were associated with 9.2 mL/min (95% CI, 5.9–12.4, *p* < 0.001) higher renal decline compared to exercise therapy. Similar results were found in the propensity score-matched paired analysis. The authors concluded that endovascular procedures for PAD are associated with clinically relevant and long-term loss of kidney function [32,123]. Prasad et al. published a meta-analysis of 20 studies that included data on patients undergoing either peripheral angiography or interventions [115]. The rates of reported AKI varied among the 15 studies, ranging from 0% to 45%, with a median of 10.0% and an average of 11.2% ± 10.0%. Seven studies with at least 200 procedures had an average incidence of 9.3% ± 2.3% and median incidence of 10.0%. An attempt to replace iodine with CO_2_ contrasts to avoid AKI was the subject of a meta-analysis by Ghumman et al. [124]. Compared with carbon dioxide (CO_2_) versus iodinated CM, CO_2_ was associated with a lower incidence of AKI (4.3% vs. 11.1%; OR 0.465, 95% CI: 0.218–0.992; *p* = 0.048). Subgroup analysis of four studies that included granular data for patients with CKD did not demonstrate lower incidence of AKI with CO_2_ (4.1% vs. 10.0%; OR 0.449, 95% CI: 0.165–1.221, *p* = 0.117). Patients undergoing CO_2_ angiography experienced a higher number of nonrenal events including limb/abdominal pain (11 vs. 0; *p* = 0.001) and nausea/vomiting (9 vs. 1; *p* = 0.006). In studies that use CO_2_ as the primary imaging agent, the average incidence of AKI remained high at 6.2%—supporting the concept that factors other than renal toxicity of iodinated CM may contribute to renal impairment following peripheral angiography [124].

It demands notice that symptomatic lower extremity PAD patients may have heterogeneous presentations ranging from mild claudication to rest pain or tissue loss. Patients with critical limb ischemia (CLI), arguably the most severe form of PAD, have a significant burden of AKI risk factors [125]. The rates of diabetes and CKD in unselected studies of the CLI population have been reported to be 30%–60% and 10%–40%, respectively [115]. Patients with CLI and tissue loss are also exposed to additional nephrotoxic agents such as antibiotic therapy which may add up to adverse effect on renal function [115]. Furthermore, patients with LE-PAD may also have an often overlooked risk factor for adverse renal outcomes, namely a high prevalence of atherosclerotic renal artery stenosis (30%–40% published prevalence) [126]. Studies by Arora et al. proved the significant role of other risk factors of AKI in patients undergoing treatment due to PAD [31,127]. The authors noted AKI rate of 11% in the endovascular arm and 12.7% in the surgical arm of the study (*p* = 0.33) [31,127].

## 7. Summary

This article hopes to summarize the principles of these approaches and help share them among the interventional community. The definition of AKI, previously known as acute renal failure, was quantified by the RIFLE criteria (risk, injury, failure, loss and end-stage) according to the KDQI group consensus and represents the latest concept adopted to ensure early detection of kidney damage [128]. CIAKI poses a serious health problem because it is a very common cause of hospital acquired AKI, linked to increased morbidity and mortality and utilizing growing healthcare resources. Further research is necessary to better understand pathophysiology of CIAKI and consequent implementation of effective prevention and therapeutic strategies (Table 5). Although many therapies have been tested to avoid CIN, the only potent preventative strategy involves aggressive fluid administration and reduction of contrast volume. Regardless of surgical technique, open or endovascular, perioperative renal injury is associated with significant morbidity, mortality and cost. Endovascular procedures always require administration of a CM, which may cause acute tubular necrosis or renal vascular embolization leading to renal ischemia and as a consequence, contribute to increased number of post-operative AKIs.

## Figures and Tables

**Table 1:** Comparison of RIFLE and KDIGO classifications.

	Serum Creatinine		Urine Output
	RIFLE	KDIGO	
Definition	SCr increase ≥ 50% within 7 days	SCr increase ≥ 0.3 mg/dL within 48 h or ≥ 50% within 7 days	UO < 0.5 mL/kg/h for 6 h
RIFLE-Risk KDIGO stage 1	SCr increase ≥ 50% or GFR decrease > 25%	SCr increase ≥ 0.3 mg/dL within 48 h or ≥ 50% within 7 days	UO < 0.5 mL/kg/h for 6 h
RIFLE-Injury KDIGO stage 2	SCr increase ≥100% or GFR decrease > 50%	SCr increase ≥ 100%	UO < 0.5 mL/kg/h for 12 h
RIFLE-Failure KDIGO stage 3	SCr increase ≥ 200% or GFR decrease >75% or SCr ≥ 4 mg/dL (with an acute rise ≥ 0.5 mg/dL)	SCr increase ≥ 200% or SCr ≥ 4 mg/dL or need RRT	UO < 0.3 mL/kg/h for 24 h or anuria for 12 h
RIFLE-Loss	Need RRT for > 4 weeks		
RIFLE-End stage	Need RRT for > 3 months		

Abbreviation: RIFLE, risk of renal failure, injury to the kidney, failure of kidney function, loss of kidney function and end-stage renal failure; KDIGO, kidney disease improving global outcome; SCr, serum creatinine; UO, urine output; GFR, glomerular filtration rate; RRT, renal replacement therapy.

**Table 2:** Incidence of AKI by anatomic location of vascular procedure [26,30,31,32,37,38].

The Type of Procedure Performed	Incidence of AKI
**Open Aortic Surgery**	
Open aortic arch repair	48%
Open type A dissection repair	45%
Open thoracic aortic aneurysm repair	34%
Open suprarenal abdominal aortic aneurysm repair	68%
Open infrarenal abdominal aortic aneurysm repair	26%
Open ruptured abdominal aortic aneurysm repair	74%–78%
**Endovascular Aortic Procedure**	
Thoracic endovascular aortic repair	9,7%
Endovascular type B dissection repair	30%
Snorkel Endovascular aortic aneurysm repair	32%
Fenestrated or branched endovascular aortic aneurysm repair	28%
Uncomplicated endovascular aortic aneurysm repair	5.5%–18%

**Table 3:** AKI in patients undergoing PCI/coronary angiography.

First Author/Year	No. of Total Patients	Definition of AKI	Numbers of Pts with AKI (%)
Hoole et al. (2009) [43]	202	Scr >25% increase from baseline at 24 h	16 (7.92)
Er et al. (2012) [44]	100	Scr ≥ 25% or ≥ 0.5 mg/dL increase from baseline at 48 h after CM exposure	26 (26)
Luo et al. (2013) [45]	205	Scr > 25% or more than 44.2 mmoL/L increase from baseline within 16 h	3 (1.46)
Igarashi et al. (2013) [46]	60	An increase in serum creatinine > 25% from baseline or an absolute increase ≥ 0.5 mg/dL within 48 h	0 (0)
Lavi et al. (2014) [47]	337	Scr ≥ 25% or > 44 mmoL/L increase from baseline within 24 h	21 (6.23)
Crimi et al. (2014) [48]	95	Scr ≥ 25% increase from baseline	13 9 (3.68)
Xu et al. (2014) [49]	200	>25% or >44.2 mmoL/L increase in serum creatinine at 16 h after PCI	7 (3.5)
Yamanaka et al. (2015) [50]	94	An increase in serum creatinine > 0.5 mg/dL or > 25% over the baseline value after 48–72 h	22 (23.4)

**Table 4:** Prospective randomized studies on AKI in patients undergoing lower extremity angiography and/or endovascular therapy for peripheral artery disease (PAD).

First Author/Year	No. of Total Patients	Definition of AKI	Type(s), Volume of CM
Rashid et al. 2004 [116]	94	Increase in SCr level > 25% or 0.5 mg/dL increase at 48-h post procedure	Mean dose of contrast in patients with AKI was 135 ± 54 mL versus 140 ± 54 mL in patients without AKI
Sandhu et al. 2006 [117]	116	Increase in SCr level > 25% or increase of > 0.5 mg/dL at 48-h post procedure	Mean dose of contrast in the NAC group was 150.9 ± 78.6 mL and in the placebo group was 125.4 ± 67.4 mL
Lawlor et al. 2007 [118]	78	Increase in SCr level > 25% or > 0.5 mg/dL at 48-h post procedure	Contrast type not specifically reported; Range of contrast use was 158 ± 165 mL
Karlsberg et al. 2010 [119]	250	Increase in SCr level ≥ 25% at 24 h	Mean dose of low osmolar contrast was 269 ± 96.79 mL and dose of iodixanol was 212 ± 94.72 mL
Karlsberg et al. 2011 [120]	253	Increase in SCr level ≥ 25% at 24 h	235 ± 99 mL(range, 38–589 mL)
Hafiz et al. 2012 [121]	320	Increase in SCr level > 25% or >0.5 mg/dL at 48-h post procedure	Median dose of contrast was 110 mL (80–150)

**Table 5:** Strategies recommended to prevent acute kidney injury (AKI) associated with vascular surgery.

Timing of Prevention	Prevention Strategy
Preoperative	Avoidance of anemia 24- to 72-h delay between intravenous administration of CM and surgery
Perioperative	Hemodynamic optimizationAvoidance of glucose variability
Intraoperative	Cold renal perfusion therapy in pararenal AAA surgery Avoidance of hemodilution Techniques to prevent procedure-related atheroembolism Limited use of intraoperative blood transfusion Individualized blood pressure management based on preoperative reference blood pressure values Remote ischemic preconditioning for selected patients
Postoperative	KDIGO bundle implementation
